# Responsible design of an AI system for health behavior change—an ethics perspective on the participatory design process of the STAR-C digital coach

**DOI:** 10.3389/fdgth.2025.1436347

**Published:** 2025-03-11

**Authors:** Helena Lindgren, Kristina Lindvall, Linda Richter-Sundberg

**Affiliations:** ^1^ Department of Computing Science, Umeå University, Umeå, Sweden; ^2^ Department of Epidemiology, Umeå University, Umeå, Sweden; ^3^ Department of Psychology, Umeå University, Umeå, Sweden

**Keywords:** participatory design, responsible artificial intelligence, human-centered artificial intelligence, health behavior change, digital coach, social norms, values, ethics

## Abstract

**Introduction:**

The increased focus on the ethical aspects of artificial intelligence (AI) follows the increased use in society of data-driven analyses of personal information collected in the use of digital applications for various purposes that the individual is often not aware of. The purpose of this study is to investigate how values and norms are transformed into design choices in a participatory design process of an AI-based digital coaching application for promoting health and to prevent cardiovascular diseases, where a variety of expertise and perspectives are represented.

**Method:**

A participatory design process was conducted engaging domain professionals and potential users in co-design workshops, interviews and observations of prototype use. The design process and outcome was analyzed from a responsible design of AI systems perspective.

**Results:**

The results include deepened understanding of the values and norms underlying health coaching applications and how an AI-based intervention could provide person-tailored support in managing conflicting norms. Further, the study contributes to increased awareness of the value of participatory design in achieving value-based design of AI systems aimed at promoting health through behavior change, and the inclusion of social norms as a design material in the process.

**Conclusion:**

It was concluded that the relationship between the anticipated future users and the organization(s) or enterprises developing and implementing the health-promoting application is directing which values are manifested in the application.

## 1 Introduction

The increasing use of artificial intelligence (AI) technology in society has led to an increasing interest in eliciting values when designing and developing AI systems in order to mitigate adversarial use of AI. Values are typically generalized and shared across societies, such as freedom, health, autonomy, and fairness, while their normative interpretations and implementations are variable, typically mirroring the local society in which they are enacted. Yet, in the current globally connected society, mobile applications promoting lifestyle changes to improve health are shared across cultures and societies, making local adaptations less viable. Furthermore, the application of participatory design principles, where individuals may influence the development and affect how such applications are designed, has transitioned into an implicit influence through the personal data provided, clicks, or through the choice to not use an application.

The main contribution of this research is a deepened understanding of the values and norms underlying health coaching applications, in particular, the STAR-C Coach application, which is being developed as part of a person-tailored digital coaching program for behavior change to promote better health and prevent cardiovascular diseases. Another contribution is an increased understanding of how the STAR-C Coach could manage and elicit different, sometimes conflicting, motives and underlying social norms to provide individual person-tailored support for changing behavior. Finally, how a participatory design process, which in itself builds on certain values such as human-centeredness and democracy, relates to the responsible AI systems design perspective is explored and discussed.

The purpose of this study is to investigate how values and norms are transformed into design choices in a participatory design process, where a variety of expertise and perspectives are represented. This was done by applying the responsible AI system design model proposed by Dignum (1) as an analytical framework to evaluate the design and development process and the developed design proposals of an AI-based digital coach for promoting behavior change to improve health. The specific research questions explored in this study are the following:

1. How are ethical aspects taken into account during design, development, and evaluation, and in the design of the behavior and functionalities of the digital solution?
2. How are the values embedded in the applied design methodologies related to the responsible design of AI systems?

The article is organized as follows. In the following section background and related work is presented. In Section 2, the participatory design process is presented and the framework is applied to evaluate the design process from the perspective of responsible design of AI systems. In Section 3, the results relating to the system’s motives and roles related to values (Section 3.1), the system’s goals related to social norms (Section 3.2), and the system’s plans and actions related to functionalities are presented (Section 3.4). The results are discussed in Section 4 and the article provides some conclusions and future research directions in Section 5.

### 1.1 Background and related work

The participatory design process in the study’s focus was conducted in collaboration between a research institution and a regional healthcare organization. The development was part of the research program STAR-C (2, 3), which built upon the Västerbotten Health Intervention program (VIP) (4, 5). The main aim was to develop a digital solution that can manage different and sometimes conflicting motives for changing behavior, including underlying social norms, and still efficiently contribute to supporting the individual’s desire and intention to change behavior. Another aim was to develop the application in a way so that the automated learning, interactive reasoning, and decision-making are transparent and sense-making to the individual.

Introducing sociotechnical systems, where values important to the workers are as equally important as technical optimizations, paved way for taking values into consideration in software design processes [e.g., (6, 7)]. The Scandinavian participatory design process started out in the Scandinavian adoption of sociotechnical systems, as illustrated by the following, expressed by Floyd et al. (6):

The underlying concept of participative system design postulated by Mumford and others is based on viewing computer applications as sociotechnical systems with multiple goals that are assessed differently by different groups according to their respective viewpoints. These different goals may be pursued and attained jointly if the values underlying them are made as explicit as possible from the start. [(6), p. 277]

Sociotechnical systems are being developed for healthcare (8), where human factors for preventing errors are taken into consideration in the development, promoting values such as *patient safety* and *patient-centered care*, and *human-centered* design is applied, where the human’s needs, desires, and interests are in focus. *Activity-centered* design is applied when the design of teamwork and work processes are conducted in conjunction with technology (9, 10) with a focus on the activity to be designed instead of the artifact (11). *Participatory design*, and participatory action research traditions where stakeholders are equally involved in and influence the development, promote values such as *democracy* and human-centeredness besides the values brought into the design process by the stakeholders (7, 10, 12). Further, in *value-centered* design practices, values and norms are elicited to develop AI systems in a responsible way, adhering to ethical standards (1, 13). Maintaining humans’ *sense of agency* and *accountability* were identified early as key mechanisms in responsible computer systems design (14). More recently, AI Design for Social Good, e.g., the UNs sustainability goals, has been coming into focus (15).

Participatory design practices, in particular the Scandinavian approaches, have maintained a strong focus on values in the design process, on maintaining the influence of stakeholders, including users, and on eliciting conflicting perspectives among stakeholders. However, its role in the current development of distributed systems across digital devices, organizations, and cultures with increasingly embedded emergent data-driven AI technologies, is being debated (16). First, in today’s global digital landscape with technology provided by multi-national enterprises, users have little or no influence on the design of technology. Second, challenges arise when the emergent behavior of AI systems cannot easily be defined, explained, or tested in order to be adjusted in a participatory approach (16). Methodologies for overcoming such obstacles have been developed and some are applied in the participatory design process presented in this article (17–19).

## 2 Materials and methods

A participatory design methodology was applied in the development of the STAR-C digital coach involving stakeholders, who are also domain experts in different fields, from the public healthcare organization and academia and future end users. In the following section, the design methodology applied in the development of STAR-C is described.

Further, the design process and its results were analyzed using the responsible AI system design methodology (1) as a framework for assessing how norms and values are elicited in the participatory design process. This is further presented in Section 2.2.

### 2.1 Design methodology

**Figure 1:**
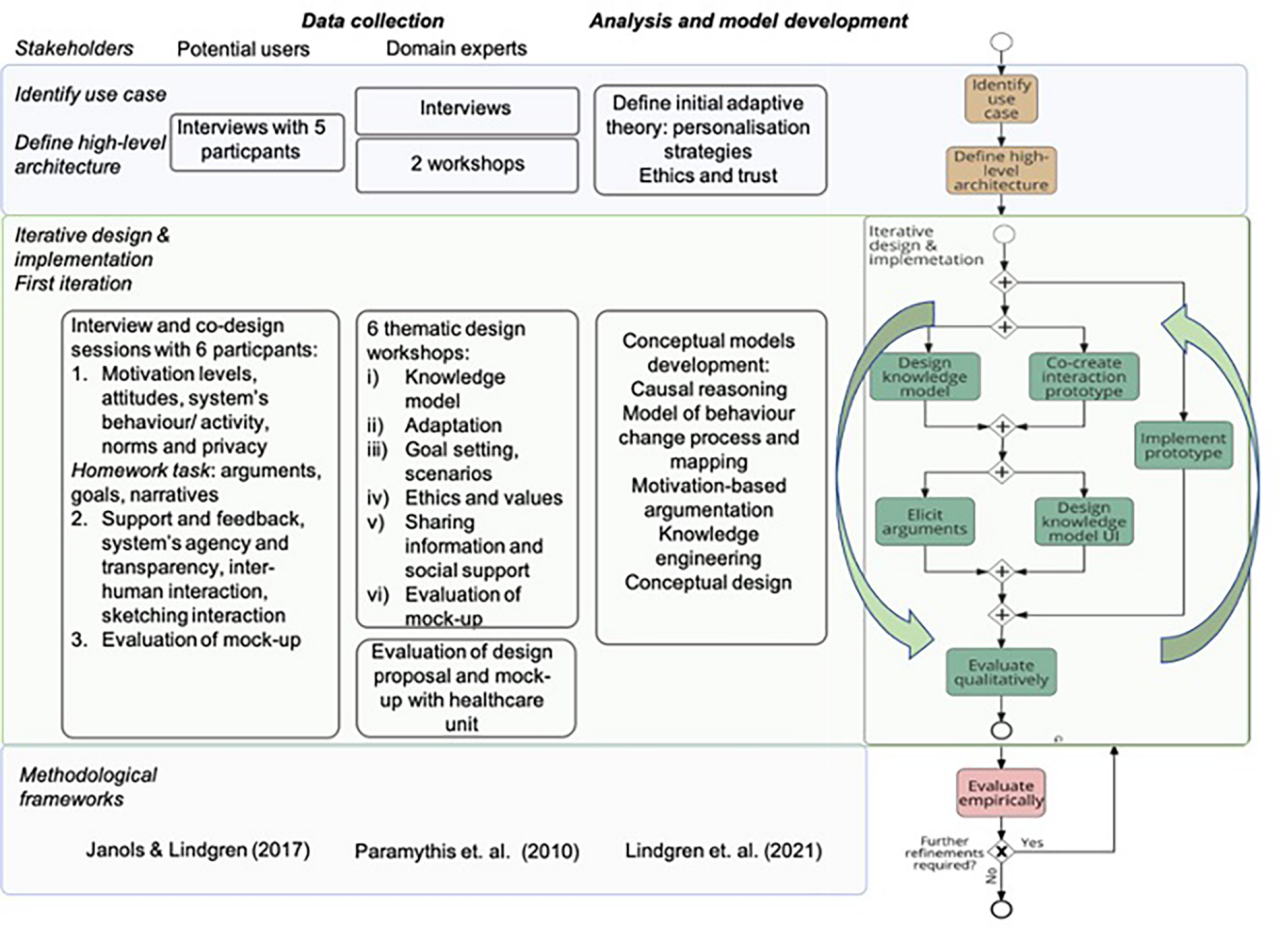
The participatory design process based on (18).

The participatory design process consisted of two major phases, as shown in Figure 1, following the general methodology presented in (18). Phase I aimed to identify the use case and define the high-level architecture, further presented in Section 2.1.1, and Phase II consisted of iterative design and implementation, further presented in Section 2.1.2.

The stakeholders involved in the design process were categorized into potential users and domain experts, as shown in Figure 1. We refer to participants representing potential users as participants or end users in the article, while participating domain experts, including healthcare professionals, are referred to as domain experts.

The content of the sessions with the domain experts followed the framework for layered interactive, adaptive systems specified in (19), with an activity-centered focus to frame the health-promoting activities in which the application could play a role (11). The co-design methodology guiding the sessions with end users was based on (17).

As visualized in Figure 1, the participatory design process presented in this article will be followed by additional iterations involving additional end users and stakeholders in the near future.

During the design and knowledge engineering process, the different roles of the participating experts and the participants representing future users were elicited in the sessions by adopting the notion of argumentation schemes (20), where design arguments are sorted based on what grounds a design proposal is presented and which values they promote. For instance, arguments supporting or contradicting a design option were identified, as were the grounds and source of an argument, e.g., evidence obtained in research, best practice, or expert opinion, or if the argument stems from a position to know. When contradictory design options were identified, these were compared and assessed during the design sessions to manage the different reasons for embedding a particular functionality or not and explore how the different perspectives could be embedded in the design.

#### 2.1.1 Design phase I

In the initial phase (upper level in Figure 1), nine domain experts were involved in the participatory design process (18). The group included representatives from two different stakeholder organizations, i.e., academia and a healthcare-providing organization, with clinical and research expertise in medicine, psychology, nursing, ethnology, social work, epidemiology, nutrition, and health economics. The researchers leading the design process provided expertise in artificial intelligence, human-computer interaction, user experience design, and medical informatics.

Following the framework for layered interactive, adaptive systems specified in (19), the relevant adaptive theory and the related values were defined. Defining the adaptive theory was done in the following way. Users’ needs and user and task models were identified and defined based on domain knowledge, theory, terminologies, and classifications specific to the health domain and core values. The methods applied were expert elicitation and validation, literature review, and ontology engineering.

The range of adaptation options was identified based on theories on persuasive systems design and behavior change systems, experts’ advice, and findings from an initial user study where five participants were interviewed. The method applied was participatory design, including interviews and focus groups. During this process, an initial selection of options was made and motivated as personalization strategies, which guided the subsequent design and development of the system (2).

#### 2.1.2 Design phase II

As a next step (middle level in Figure 1), the first iteration of design and implementation was conducted involving domain experts and future users in two parallel but intertwined participatory design processes. Data generated by each session were analyzed, fused with earlier results, and visualized partly through changes in the design of the mock-ups. Design proposals and reflections were communicated across the groups between sessions, partly through the changes in the design of the mock-ups used for exemplifying design proposals.

The development of the conceptual design, the model of the behavior change process and its mapping, the engineering of knowledge, and the argument-based causal reasoning were informed by the design sessions and embedded in the STAR-C Coach. They formed the building blocks for the AI system analyzed in this study and are listed in Figure 1. The behavior change process model is presented in (21) and the initial work on the argument-based causal reasoning in (22).

Six co-design workshop sessions were organized with the domain experts, involving researchers and two representatives from the regional healthcare organization. These were followed by a session focusing on the evaluation of a design proposal and mock-up which was conducted in a team with five participants from the unit governing the development of healthcare services in the regional healthcare organization.

Six persons who had taken part in VIP participated in co-design sessions and interviews, representing future users. Recruitment was done by sending invitation letters to a randomly selected group of 40 persons who participated in VIP in 2019, with information about the study and indicating that a researcher would call shortly. Six participants agreed to participate, two women, aged 42 and 62 years, and four men, between 42 and 62 years (Table 1). Four of these participants participated in three sessions, and two participated in one session. The sessions were mainly organized individually, using Zoom, following COVID-19 restrictions. One of the last sessions was a joint session with two of the participants. The sessions were recorded and notes were taken. The participants were also given a task to do between sessions. The task consisted of the participants reflecting on their views on setting goals for lifestyle changes relevant to them, opportunities to change habits with a digital coaching tool, moments of self-reflection, and how such moments could be supported by a digital application.

**Table 1:** Participants in the study. Participants marked with an asterisk (*) participated in the first session only.

P	Gender	Age	Technology literacy	Readiness for change	Targeted behaviors
1	F	42	High	Preparation	Nutrition, physical activity
2	F	62	Medium	Preparation	Nutrition, physical activity, stress
3*	M	43	Medium	Maintenance	Physical activity, nutrition
4*	M	62	Medium	Maintenance	Physical activity, nutrition, stress
5	M	62	Medium	Maintenance	Physical activity, nutrition, stress
6	M	42	Low	Contemplation	Smoking

The habits the participants wanted to change typically related to more than one of the domains covered by the study (Table 1). Based on the interviews, the participants’ level of technology literacy and readiness for change based on the Transtheoretical Model of Chance (TTM) (23) were assessed.

### 2.2 The responsible AI system design methodology as framework

**Figure 2:**
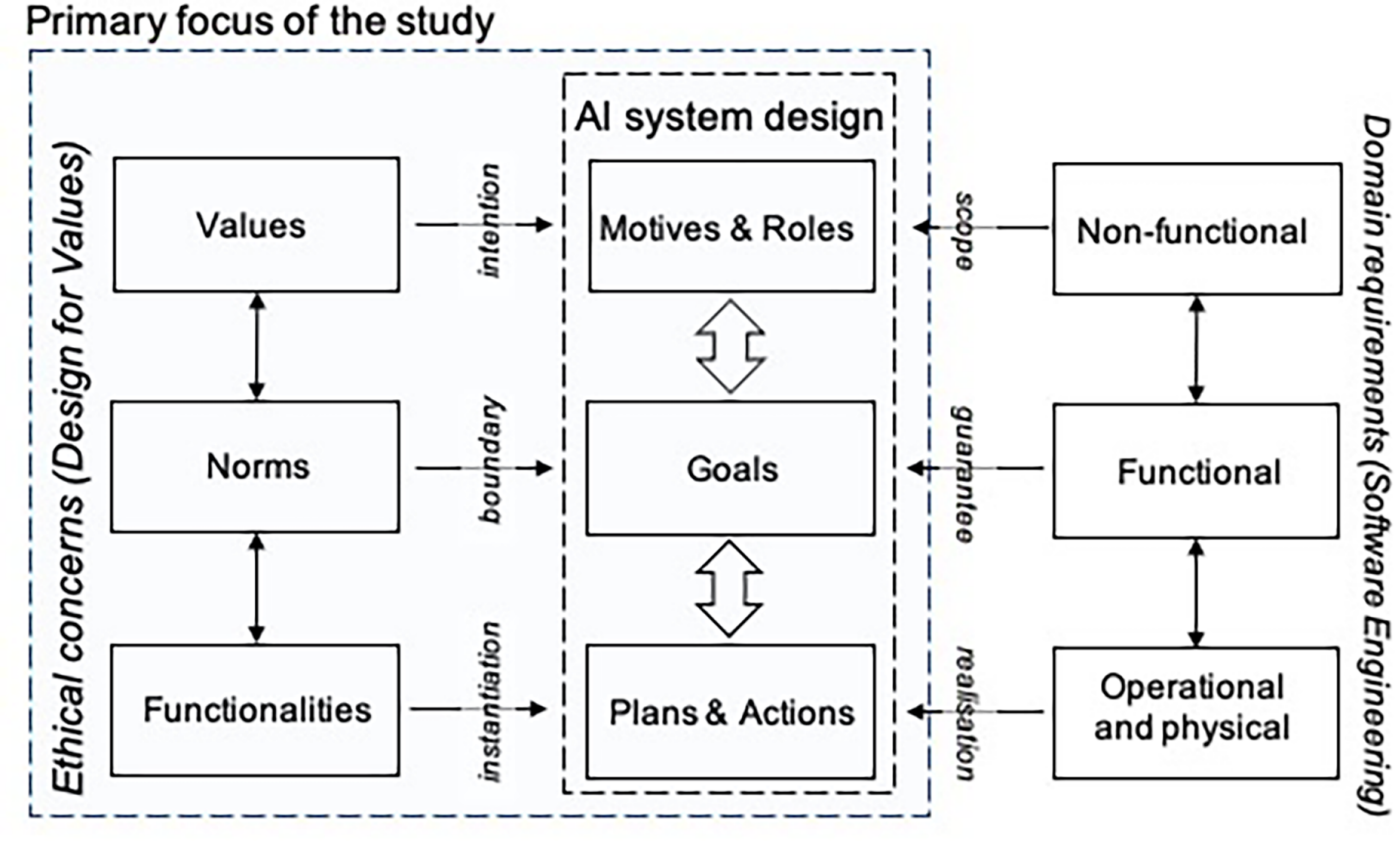
A model for responsible AI design presented by Dignum (1).

The design of the STAR-C Coach was analyzed using the responsible AI system design methodology as a framework (1) (Figure 2). The framework is proposed as a means for eliciting ethical aspects relating to the values and norms embedded in the design process that affect design choices in AI systems. Ethics refers to moral principles that guide and govern behavior. *Values* are viewed as generalized, commonly shared across populations and societies, while the implementation of the values through *norms* are specific to cultures and local societies. The values and norms, and how the norms are translated into functionalities, influence AI system design in terms of the motives and roles of the AI system, and its goals, plans, and actions (Figure 2). In this article, we focus on the ethical concerns and the AI system design and defer the software engineering perspective to future work. Further, the design process of the STAR-C Coach is discussed in Section 4 from the responsible design perspective to assess the utility of applying the participatory design methodology for responsible AI system design.

During the first phase and the co-design sessions in Phase II, various aspects regarding values and social norms were brought up by the participants. The specified values and norms and their relation to health applications in general, and specifically to the STAR-C coach application, were discussed during the co-design sessions with six participants representing potential future end users (1). These discussions were conducted as part of the participatory design process. The data collected and the design proposals were analyzed using the responsible AI system design methodology as a framework (1) (Figure 2).

The specified norms and values and the AI system’s related motives and goals were modeled using an argumentation framework and embedded in the design of the digital coach. The results were exemplified and evaluated by the domain experts and the participants.

The values and norms that were discussed among participants in the design process were analyzed and connected to the system’s goals, plans, and activities to be conducted together with those of the user, such as setting goals, as were the requirements that emerged through the process.

The results of the analysis were evaluated by the expert group during a workshop to verify the interpretation of the data collected during the design process (thematic workshop iv), as shown in Figure 1. Adjustments were made based on the results.

## 3 Results

The results are organized based on the model for responsible design of AI systems (1) (Figure 2), with a focus on ethical concerns and AI system design. In the following section, the results regarding the system’s *motives* and *roles* related to *values* are presented. The system’s *goals* related to *social norms* are presented in Section 3.2, and the results regarding the system’s *plans* and *actions* related to *functionalities* are presented in Section 3.4.

### 3.1 The system’s motives and roles in relation to values

The participating experts agreed that the purpose of the application is to support the citizen in their activities to prevent cardiovascular diseases by promoting the maintenance of healthy behavior and promoting behavior change towards healthy behavior in the domains of physical activity, tobacco use, alcohol consumption, nutrition, and stress. The role of the application is to function as a digital coach for the citizen between participation in VIP every 10th year. Support for changing behavior between the health check-ups was expressed as something missing by the participants, who also indicated that a digital tool could serve this purpose.

The application is being developed as a complement to the motivational health conversation that the nurses have with citizens in conjunction with health check-ups in primary care (24). The regional healthcare-providing organization is consequently a key stakeholder in the development of the application. A 35-year collaboration between research and regional healthcare to promote lifestyle changes for better health has built the medical evidence-based scientific foundation for the development of the application. As a consequence, researchers from different scientific domains are also stakeholders in this process. A few of them also have employment in or affiliations with both academia and healthcare organizations.

The potential users and experts emphasized that the support provided by the application should be person-tailored and its relevance optimized for the person.

The digital coach could be introduced by a nurse, and the initial goal setting would ideally be performed based on the health conversation (motivational interviewing) with the nurse. The citizen could also on their own initiative begin using the application. It was discussed whether the digital coach would be provided by the healthcare organization, and as such, comply with the regulations concerning documenting health interventions, storing patient data, etc., or be an application owned and controlled by the user, in particular, in terms of ownership of personal information. Considering that the user will typically not be a patient in the years between the health check-ups, the initial assumption was made that the owner of the application, and the personal data that it is collecting, will be the user. The user decides if and when to share information with healthcare representatives or others.

It was decided by the experts to not involve an enterprise in the design process in order to focus on values during the development process other than commercial values.

#### 3.1.1 Values

The values that the stakeholders promote are the following. The regional healthcare-providing organization is responsible for providing healthcare based on evidence-based medical knowledge, best practices, and ethical regulations in order to improve health in society in a cost-efficient way considering that healthcare in the context of our study is tax-funded. Healthcare should ideally be equally accessible to all, regardless of where they live and their age, socio-economic conditions, or gender. A particular challenge is that the region is geographically large and sparsely Populated, often with long distances to services and specialist healthcare, in particular. As a consequence, the provision of healthcare is directed by the following key values: *evidence-based medical and health knowledge* to optimize *quality* and *equality* of care, *fairness* in accessibility to care, and *health-economics* to optimize the resource allocation and distribution, and consequently, value for society.

The following values were identified and discussed among the participating domain experts from a user’s perspective, which can also be viewed as the needs of an individual: *autonomy*, *competence*, *relatedness*, *privacy*, *trust*, physical and emotional *wellbeing*, and *self-determination*. The impact a person’s social environment has on behavior and motivation was also considered an important factor, and to some extent, it can be also considered a value. This impact may have a positive or negative effect on healthy behavior, which will be further explored in the next section. A digital coach displaying social behavior was also expected to provide a sense of *companionship*.

The values that the researchers and practitioners contributed were, besides those already mentioned, related to how humans in society relate to each other and to technology. Values such as *empathy*, *niceness*, *competition*, *social influence*, *safety*, *experience*, and how humans should *be in control of technology* were discussed.

To summarize, the developed adaptive behavior of the digital coach was expected to provide the user experience of *control, sense of agency, competence, relatedness, trust* and a sense of *companionship* in relation to using the system and pursuing the objectives of desired activities. These form the basic set of values defined for the development of the STAR-C coach.

### 3.2 The AI system’s goals in relation to social norms

Values can have different normative interpretations in different social and cultural contexts. In our study, the social and cultural context is the regional society in which the digital coach is being developed, and the outcome will reflect the stakeholders and expertise areas involved in the process. Consequently, the social norms presented in this section are specific for this development context. Some examples of norms that were elicited in the sessions during the design process are listed in Table 2 with the related values and goals of the system. Some norms are motivated by or reinforced by society, and some are internalized in an individual as their own. These will be further discussed in this section.

**Table 2:** Overview of identified norms, their motivation, related AI system’s goals, and functional requirements. Norms marked with an asterisk (*) embed conflicting viewpoints.

n	Norm	Value	System’s motive	System’s goal
1	Each individual decides about and is responsible for their behaviors	Autonomy, self-determination	Increase and support both extrinsic and intrinsic motivation, adapt to and support the individual’s goals	Assist in goal-setting based on the human’s readiness for change, support through person-tailored motivations for change
2	Healthcare should be based on established medical and health knowledge as far as it exists, and/or on health-economic values	Wellbeing, health, and trust; justification of health-promoting interventions with the aim to change someone’s behavior	Base risk assessments and advice on medical and health knowledge	Provide personally and medically relevant, transparent, motivating risk assessments and advice
3	One should be physically active	Physical and mental health and wellbeing	Increase physical health and wellbeing and knowledge about this	Monitor and increase support, maintenance of physical activity, and knowledge of the benefits thereof
4	One should eat healthily	Prevent diseases and increased cost for society	Improve nutrition habits and increase knowledge about the benefits	Monitor and support changes in nutrition habits and increase knowledge of the benefits thereof
5	One should stop using tobacco	Prevent diseases and increased cost for society	Support ending the use of tobacco, increase knowledge about the benefits	Monitor and support stopping the use of tobacco and increase knowledge of the benefits
6	One should decrease alcohol intake	Prevent diseases, reduced wellbeing, and increased cost for society	Reduce alcohol intake and increase knowledge about the benefits	Monitor and support decreased use of alcohol and increase the knowledge of the benefits
7*	(a) One should work hard and be successful; (b) one should be kind to oneself	(a) Economic and social status: competence, autonomy, and relatedness; (b) rest and recovery	Prevent negative consequences by striving for manageable stress levels and sufficient rest and recovery	Monitor and support a healthy balance between work and rest
8	The human is in control of technology	Autonomy, self-determination, competence, and privacy	Support a Sense of Agency (SoA), shared intention, and trust	Provide transparency, explainability, and control mechanisms of its behavior
9*	(a) One should present oneself in social media; (b) one should *not* present oneself in social media	Relatedness, privacy: position in a social context, (a) partly by presenting oneself in social media, or (b) without presenting oneself in social media	Communicate a self-image to the person and potential others	A communicable personalized visualization of the behavior change progress
10	Empathy and niceness should be embedded and expressed in social situations	Emotional and social wellbeing	Mediate a sense of comfort, and likability	Act nicely and empathetically
11	One should do things together with others	Relatedness: to nurture and be motivated by social relationships	Acknowledge and support social relationships	Use as motivating arguments, encourage social activities

#### 3.2.1 Norms, autonomy, and self-determination

Looking after one’s health can be considered a societal norm, by which individuals are expected to comply. The individual’s own explicit desire, motivation, and decision to aim to improve their health through preventive actions (n1 in Table 2) was extensively discussed, since it is considered to be at the core of a health intervention and a key requirement in this work to ensure self-determination and autonomy, and also to have the potential to be successful. Yet, the difficulty in changing behavior and sustaining a change of behavior was discussed, in particular, how to guide the individual to formulate small easy-to-achieve goals (“baby-step” goals), and how to motivate the continuation of the changed behavior. However, the situations in which the individual is making decisions about whether or not to do the planned activity provide barriers that may prevent the person from following the plan. Examples that were mentioned during the sessions with the experts were contextual factors such as weather, socioeconomic situation, and physical location.

#### 3.2.2 Norms motivated by evidence-based medical knowledge and best practice knowledge

Some of the social norms (n) in society are embedded in the provision of preventive healthcare, supported by evidence-based medicine (n2 in Table 2).

Person-tailored advice based on evidence-based and professional knowledge was considered one basic requirement for promoting health and justifying the promotion of norms related to health behavior and lifestyle changes (n3–n7).

While the social norm to work hard and be successful (n7) may lead to high pressure to achieve, leading in turn to an increased risk of stress-related medical conditions, it was emphasized that research also shows the importance of balancing work and recovery activities.

Being physically active is a norm well-founded in research as a foundation for both physical and mental health, while sedentary behavior leads to an increased risk of cardiovascular diseases.

Healthy nutrition habits help prevent cardiovascular diseases. Related norms are to maintain healthy food and drinking habits. Smoking and alcohol intake were viewed by the participating experts as problematic, since such behaviors have a negative impact on health and increase cost for society, partly in terms of healthcare. However, it is becoming more acceptable to drink alcohol on weekdays, which was previously not considered acceptable. This is an example of norms that change over time. There are also changes in what is considered to be risky consumption of alcohol.

#### 3.2.3 Social norms

There are many, often conflicting, norms, attitudes and expectations in society related to physical activity (n3), eating (n4) and drinking behaviors, and consumption of toxic substances such as tobacco (n5) and alcohol (n6), and not all norms are supported by research findings or medical and health knowledge. Often, such norms relate to appearance, looks, and status in a social context, where some behaviors are considered “better” in some sense than others, and consequently, impose judgement on the person. It was extensively discussed how the system may negatively impact self-image if obesity is a problem, and how to not mediate judgment. It was concluded that the system should not embed weight as a parameter and instead focus on factors and behaviors that are more easy to change.

Social media is further reinforcing norms, attitudes, and implicit and explicit judgements, which were also discussed and studied in relation to STAR-C (n9). Society embeds norms relating to how to present oneself in social communities, online, and in the physical world, which also may cause stress (n9).

Social actors are expected to be empathetic among other actors in social situations (n10) and the experts emphasized how the system should avoid causing guilt or shame, e.g., when failing to comply with what is expected by themselves and/or others related to weight, physical exercise, etc. The STAR-C coach’s goals should provide knowledge-based support for increasing physical activity, and for reducing unhealthy behaviors such as sedentary behavior, stress, unhealthy eating habits, smoking, and alcohol consumption, however, at the same time, avoid imposing negative emotions such as shame and sense of failure. How the system could be designed to mediate a positive and motivating experience, even when providing fearful facts, was extensively discussed among the experts. How the behavior change progress is communicated to the individual was consequently considered very important. Moreover, it was also discussed how the system should communicate a sense of empathy, a kind of understanding of the individual’s situation (n10).

#### 3.2.4 Norms relating to technology

There are emerging, often conflicting, norms relating to how technology should function in relation to humans. For instance, that the human should be in control of technology is a frequently repeated norm (n8) that was discussed among the experts on the one hand, but on the other hand, the human is also expected to be always digitally connected and available through a smartphone, and a digital coach was expected by participants to need to be proactive to some extent to be useful. Sense of control and SoA (25) are factors explored in human–AI interaction research and were found to be reduced in situations where a computer system is introduced in a collaborative setting (26). The STAR-C coach’s goals consequently include promoting SoA and a shared intention (n8). Related requirements are explainability and transparency, and mechanisms to control the behaviors of the AI system.

During the sessions with the experts, it was discussed that people are also expected to communicate information about themselves through social media, and it was pointed out that attitudes towards this among the VIP participants were as yet unknown. An emerging conflicting norm is to not share information and instead keep information private. Another norm is to pay attention to the situation and not the phone when in a situation with other people. As a consequence, introducing yet another digital application in a situation in which a person is already overwhelmed with digital tools may be counter-productive as a means to promote health. The interaction with the system should then at least minimize the amount of cognitive load that is required to use the tool and adapt to a situation.

#### 3.2.5 Future users’ perspectives on norms

The participants agreed with the health-related norms n1–7 listed in Table 2 and did not view that a health coach application would add undesired pressure to be successful.

A general trust was expressed towards health application providers regarding willingness to share information and to the healthcare organization in particular to achieve a greater use benefit; however, transparency and explainability were desired (n8). Commercial applications were less trusted regarding their health-related content compared to an application provided by their healthcare organization. Sharing on social media was undesired by all participants (n9). Nice behavior (n10) was seen as less relevant for a coach application taking on the role of a digital coach delivering hard facts. Yet, having a character representing the coach was perceived as interesting, but was not expected to increase motivation in most of the participants, except for two (P5, P6), who saw benefits in having a character intervening in moments when support is needed. Doing things together with others (n11) was seen by two of the participants as key to increase motivation to change behavior.

Examples were shown to the participants of how the AI system could provide information about norms underlying the AI system’s motives. The examples were received positively and triggered thoughts on norms. In particular, norms relating to visual appearance were brought up. The barriers to using public venues for exercise when overweight were discussed, and how to motivate behavior change under such conditions, when a person starts out expecting to fail. A norm relating to obesity and overweight was also discussed by the expert team but was not included among the norms that they considered that the AI system could use as a basis since the stigma around this and the difficulties of doing something about this were considered too difficult a problem to be managed by the AI system. Yet, the participants brought up losing weight as a main motive for behavior change relating to nutrition and physical activity, which illuminates its importance to potential users.

#### 3.2.6 Norms translated into functionalities

The AI system’s goals and motives are organized into activities and conducted at some levels in collaboration with the user. Consequently, the defined general functionalities that enact the values and norms can be described and summarized in terms of the following personalization strategies identified during the first phase of the design process (Figure 1) (2):

1. Multi-modally engaging goal setting to identify the individual’s desire and intention to change behavior (n1).
2. Embedded relevant evidence-based knowledge as a base for generating, communicating, and reasoning with personalized information about risks and, potentially fearful, facts (n2; n3–n7).
3. Management of privacy including the possibility to share content (n8, n9).
4. Assessment of progress in interaction with the user and personalized interactive visualization of progress, or non-progress, feedback, and rewards—to increase motivation and support the construction of a positive self-image (n1, n9).
5. Avatar as coach to mediate the social and emotional support (n10–n11).

These personalization strategies conform to design principles for behavior change systems defined in (27) (tailoring, dialogue support, social support, credibility, self-monitoring, simulation of desired goals, or connection between causes and effects). They also cover the values and social norms identified in the design process of the STAR-C coach (Table 2).

### 3.3 The participants’ perspectives on the AI system’s role, trust, privacy, social support, and motivation

The role of the coach application was viewed as primarily a coach, which sometimes delivers also hard truths about progress and predictions based on medical and health knowledge. If the application is provided by the healthcare organization, the messages and assessments would feel more reliable and trustworthy than those delivered by commercial applications. One participant (P4) also indicated that proactive behavior pushing for behavior change would be acceptable from an application provided by healthcare, but not from a commercial source.

The participants had no problem giving away their personal data, in particular, if the healthcare-providing organization was the receiver. Moreover, they saw only benefits if their patient data were linked to the digital health coach.

The participants did not want to share information on social media. One of them (P2) viewed posts on social media as bragging, and consequently, not complying with norms on how to behave. Another participant (P5) did not want to expose personal information to people. One participant (P1) would consider sharing in a small group of supporting people with the same level of ambition and focus on their goal to change behavior, but not on social media.

The importance of social support in gaining motivation and get going was emphasized by two participants (P1, P2). P2 would like to have a way to get to know others in the region, within visiting distance, to do some activities together, and share the efforts to change behavior. Living in rural areas provides some barriers to meeting others and participating in activities.

In the second step of the co-design process, ideas were explored regarding how to engage users through the application across the region in collective activities, which had been elicited both in workshops with domain experts and in the earlier interviews with the participants. This was done based on earlier views on sharing information, privacy, and anonymity, and also views on the importance of social support. Mock-up examples of use scenarios were developed and discussed during the sessions, forming a base for triggering new ideas and scenarios. As a result, a design proposal was also developed regarding how users can be anonymous while finding others in compatible situations with similar levels of ambition and barriers to changing behavior. One idea is that a person can choose to support others, by sending messages as explicit “nudges,” which could serve as the desired social support, or the “*social control*” that they needed, as two participants described it (P1, P6). The wish to be of help and support others was expressed, with sending nudges seen as one way to do so.

A wish that was expressed by P1 is that then the AI system coach could propose who to nudge, without revealing the identity if the person did not want this. Another wish was that the healthcare-providing organization could then target tailored interventions to sub-groups in the region who have collectively defined their level of challenge relating to one of the health domains.

#### 3.3.1 Mobilizing motivation

P1 indicated that the main challenge was to mobilize motivation to adhere to the goals that had been set up and to not expect too much progress too soon. The main obstacle related to nutrition was the strong desire to eat the food and sweet things that they desired.

To be able to access an overview of the progress was seen as highly valuable and one person (P5) indicated that the overview of accomplishments over time is the main reward and motivator. This was important to the extent that when the device measuring time and distance did not work or was not charged, he would not do the planned exercise. Another participant (P5) explained how the star visualizing the results of the health checkup at the age of 50 was perfect, while at the age of 60, the star was bad for all parameters. This was a warning sign that gave him the motivation to change his lifestyle. Now, a few years later, he would like to create a new star to see how much it has improved.

One participant (P5) wanted to have automated logging of data since he thought that manual registration would not be done. In addition, he did not want reminders. Manual logging of information was not seen as an obstacle by others. One person (P1) did not need the freedom to avoid answering some questions, as she assumed that mandatory questions would be necessary for the application to conduct analyses. However, it was considered valuable by all that the application should explain the basis for assessments, e.g., in the case of predictions based on behavior change trajectories.

One participant (P5) highlighted the need for support at the moment when support is needed, e.g., when the temptation is high in a store to buy unhealthy food, or when stress levels increase to remember to relax and breathe. A type of companion could be used, e.g., a dog that barks to remind the user to go for a walk, or a watchdog in the store that reacts if unhealthy food is picked.

A problem with existing commercial applications that was mentioned is that they sometimes embed norms on what constitutes particular activities. One example mentioned by P1 is that walking is not registered as walking if one walks too slow, e.g., due to disability or being overweight. One older participant (P2) would like to have support to handle pain conditions that prevent her from being physically active. She also described a conflict in balancing the need for rest and recovery due to a stressful work situation with increasing physical activities, in addition to taking pain conditions into account.

In most participants’ view (P1, P2, and P5), digital rewards did not help to increase motivation, and instead, hard facts, with proactive assessments and suggestions from the coach application, in combination with social support in the form of someone checking in once a week (P1), or a small support group, would help (P1, P2). Moreover, one participant (P1) mentioned that she would like to be able to conduct physical activities that were considered to be fun, which would increase motivation, instead of having to do boring activities such as taking walks.

### 3.4 The AI system’s plans and actions in relation to functionalities

**Figure 3:**
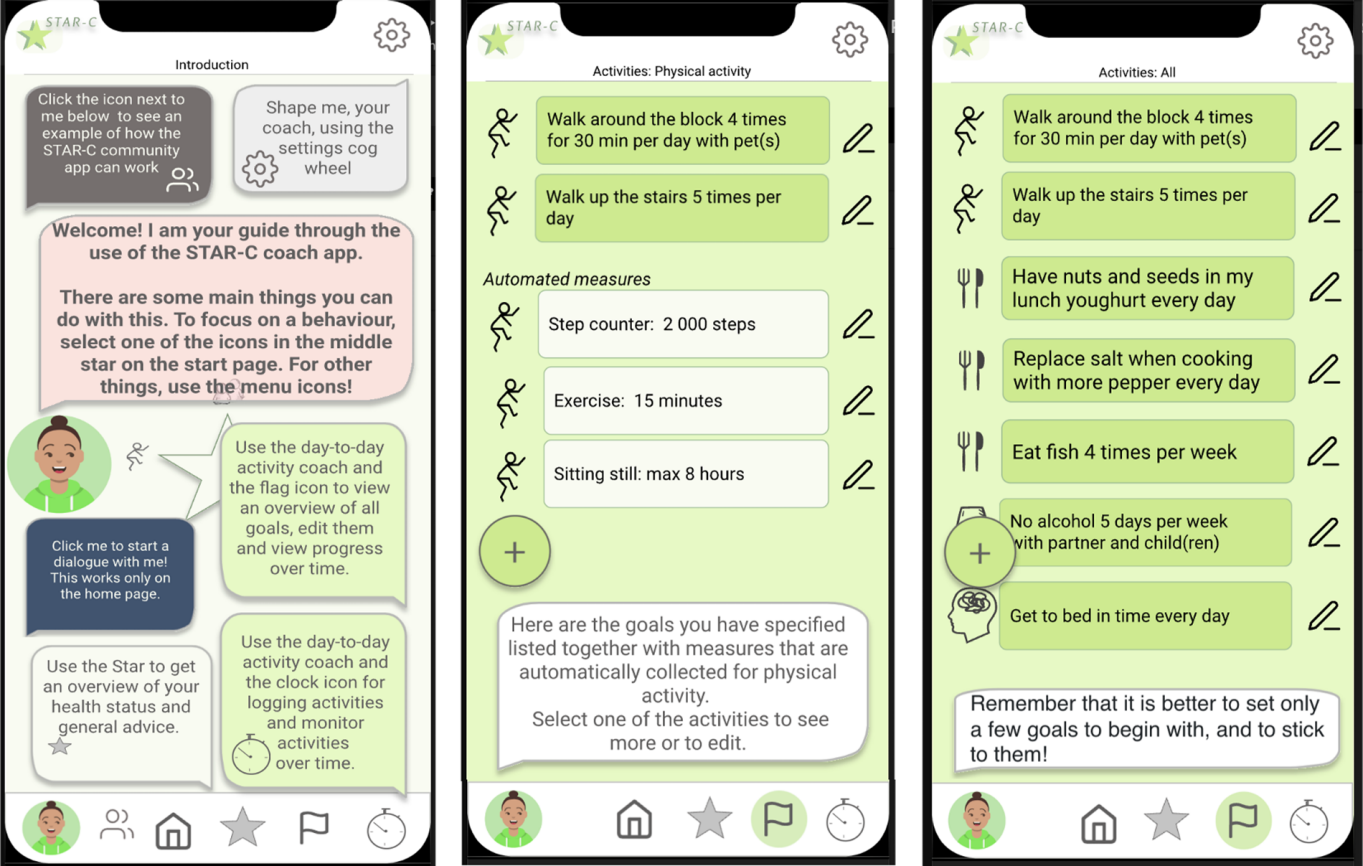
Overview of the functionalities and examples of a checklist and goals related to physical activity.

**Figure 4:**
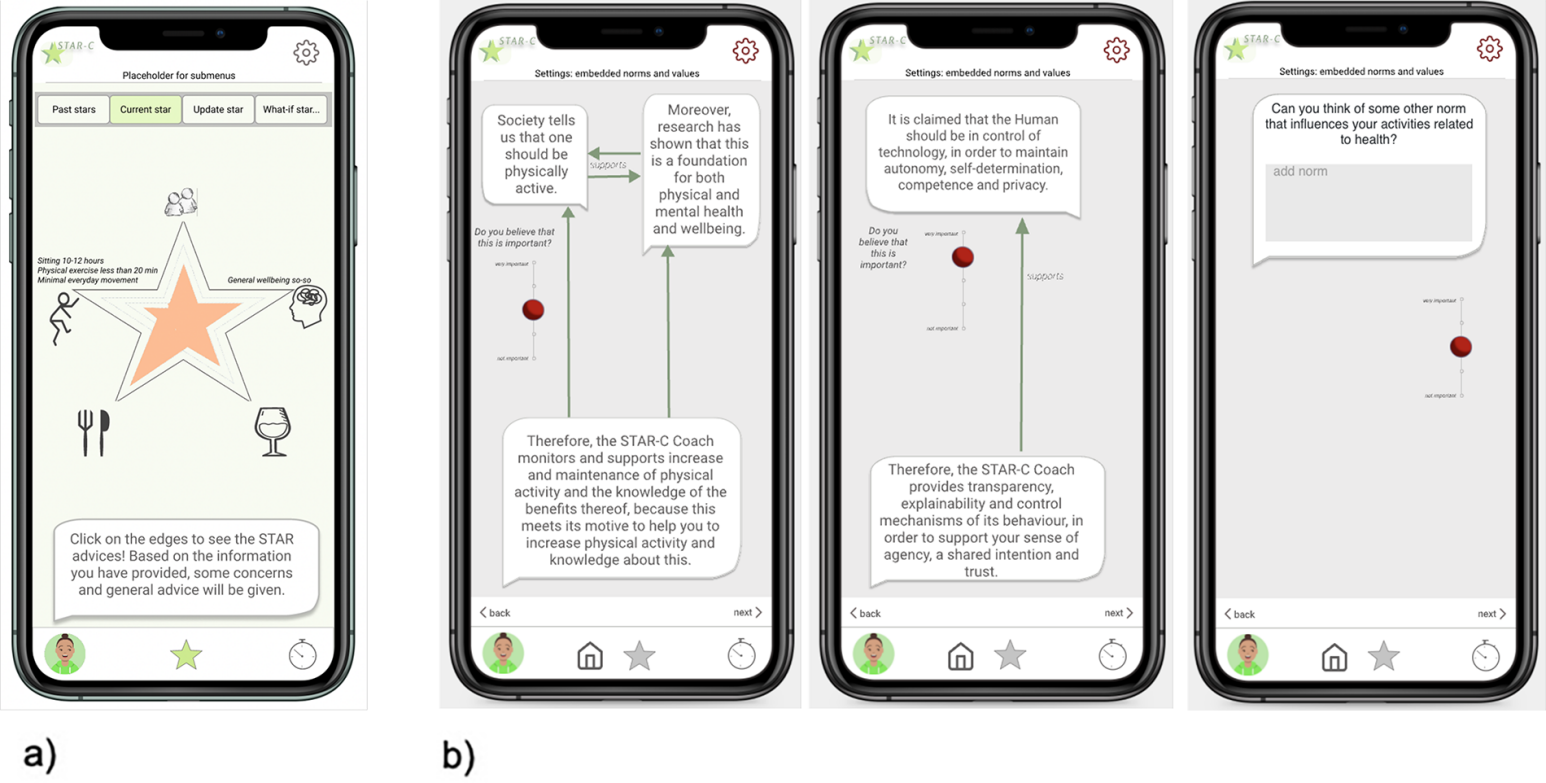
**(a)** Mock-up star profile and **(b)** a mock-up shown to participants of how the norms and the coach agent’s goals were related.

Based on the analysis and findings from the user study, an early design proposal for the AI system was further developed into a conceptual design, mock-ups, and interactive prototype. These were evaluated by participants, experts, and a team at the regional healthcare organization responsible for developing new digital instruments for primary care as the final design activity in the first iteration described in Figure 1. The interactive mock-up is illustrated in Figures 3 and 4.

The general functionalities identified previously were (i) interactive communication and visualization of behavior change progress, (ii) person-tailored risk assessments and advice based on evidence-based medical and practice knowledge, (iii) goal setting, (iv) avatar as a coach for promoting social support, and (v) management of privacy and sharing of information.

These were organized into the following three main modules in the resulting prototype: (i) *the Star*: representing the holistic view of one’s health (Figure 4a); (ii) *the day-to-day view* listing the selected daily or weekly activities (Figure 3); and (iii) *settings* including shaping the coach and presentation of underlying social norms and values (exemplified in Figure 4b). In addition, (iv) a chatbot view can be accessed as a communication channel with the digital coach, and a fifth module was included based on the co-design results: (v) the STAR-C community. While the STAR-C community module was perceived as particularly interesting by some of the participants and some of the regional healthcare representatives, legal and responsibility aspects were raised by other representatives of the healthcare organization. In particular, the responsibility to monitor the contents of interactions to ensure that they adhere to social norms and legal aspects was discussed.

The AI system’s plans and actions are motivated by the system’s motives and goals, and are tailored to the individual’s motives, goals, and their level of readiness to change behavior. The motives include supporting the individual in improving physical, social, and emotional wellbeing and increasing knowledge about the benefits of health-promoting activities.

## 4 Discussion

A combination of methods was applied in the study and explored from the perspective of eliciting values and norms in the design process. These are discussed in the following section, followed by a discussion of the results in Section 4.2, and the strengths and limitations of the study in Section 4.3.

### 4.1 Value elicitation through the participatory design methodology

The participatory design methodology adopted in the development was selected based on the expected values of participation, e.g., (i) allowing stakeholders to influence the shaping of their tools, (ii) highlighting different potentially conflicting perspectives to reach agreements on prioritized design choices, (iii) the focus on values, and (iv) the focus on the process of shaping the clinical work routines, research processes, and the citizen’s everyday activities contributing to improving their health, rather than an artifact as the end product (7). Consequently, the participatory design approach ensures that values are taken into consideration in the design process and is consequently aligned with the purpose of responsible design of AI systems. Analyzing the participatory design process from the perspective of the responsible AI design framework (1) provided (i) increased awareness of the value of participatory design approaches for responsible AI system design, which has also been discussed elsewhere recently (28); and (ii) the inclusion of social norms as *design material* in the process, similar to design material that can be modeled such as personal data, medical knowledge, and guidelines relating to health.

Healthcare is a domain that is governed by ethical regulations, both regarding healthcare provision and regarding how the tax money funding the services is used, and consequently, *responsibility* and *accountability* are embedded values, which are values emphasized in relation to guidelines and recent regulations on AI systems (1). This may help explain the difference that was seen between the attitudes expressed by the domain expert group and the group of potential future users. While the expert group, partly representing the healthcare-providing organization, took a cautious approach to how the coach application should address the users, the user representatives wished for hard facts and not necessarily only pleasant information. One example is how to put forward the need to reduce weight. While the potential users found this to be one of the main motivating factors for changing behavior, representatives for the healthcare-providing organization were cautious as to how to address this so as to not give the user too much hope that a coach application can help address their obesity. This is in contrast to how commercial health applications advertise that paying additional fees would result in reducing weight. One widely used example is the nutrition app MyFitnessPal,^1^ promoted by the Apple Health app,^2^ which provides overviews of a person’s data if they pay a fee for the premium version, which in turn, is promoted by the argument that certain percentage of the users reduced their body weight.

There is a multitude of health and fitness applications developed for particular purposes such as increasing physical exercise, improving nutrition and sleep habits, and monitoring and reducing alcohol and tobacco use (29, 30). However, there are only a few applications that adopt a more holistic perspective by taking a number of domains of interest into account in a similar way as the STAR-C coach. A review of applications targeting cardiovascular disease prevention in the same way as STAR-C does showed that only 8% targeted more than one lifestyle domain (31). One example in the review is an app targeting physical activity, nutrition, and obesity (32). The study showed that participants with under to normal body weight appreciated and used the intervention, while participants with overweight and obesity did not, since it reinforced their negative self-image. This example illustrates the challenge of positively promoting lifestyle changes. Commercial applications typically function by importing and sharing data with other health applications, targeting particular domains to integrate more domains into their applications (e.g., Apple Health).

Health and fitness applications can be categorized as recommender systems, behavior change systems, or persuasive technology, depending on which contexts they are developed. Some may take ethical considerations into account (27, 33) relating to being transparent, not deceitful, and promoting and supporting only the user’s intention and desire for changing behavior, which are requirements for behavior change systems as defined by Oinas-Kukkonen and Harjumaa (27). Whether the individual has the right to be *not* exposed to nudges aiming to change their behavior is debated from ethical and philosophical perspectives (34, 35). Tengland discusses the balance between behavior change, its ethically problematic foundation (33), and empowerment. The balance between guiding the individual to pursue their own aim to change behavior and reinforcing the behavior desired by society and the healthcare organization requires a professional awareness of the clinician’s own attitude and influence (24).

We argue that the fundamental basis for such differences and conflicts in perspectives is the observation also made in this study: the differences in the *relationship* between the future users and the team and organizations developing the behavior change system. This relationship has been clearly defined as a part of the participatory design process in this study, where the future users are primarily *citizens* of the sparsely populated region for which the regional healthcare-providing publicly funded organization is responsible when the citizen shifts their role to become a *patient*. The participating academic institution, also funded by tax money, has the responsibility to educate, provide new knowledge, and collaborate with the surrounding society and its citizens. These relationships ensure a shared interest in health as “lived” and nurtured in this particular community.

The contrasting relationship is the one between commercial health and fitness app producers and the consumers of health technology. This consumer-producer relationship is built on the value exchange between the two: the consumer gains value in terms of interpretations of their health data in exchange for personal data, information, and money. The rationale for the producer is the higher the number of paying customers, the more revenue for the enterprise.

The purpose of the STAR-C coach application is to a certain extent the same: the user gains an interpretation of their health data in exchange for data and, indirectly, tax money. However, the rationale for the participating healthcare-providing organization is different: the more active users, the fewer the potential patients and consequently, a decreased cost for society.

What is then the fundamental difference between the support provided by the commercial health and fitness applications, and the potential support provided by the STAR-C coach application? The major difference resides in the socio-cultural context in which the STAR-C coach application has emerged. The support that the STAR-C coach application can provide will be based on evidence-based knowledge and best practices developed in the region, which has already supported a shift in behaviors among citizens in the region towards healthier habits and improved health (4). This intervention is one of several complementary interventions that the citizens have access to, which together makes a holistic effort to address medical and health issues in the broader population, taking into account the particular challenges that come with the fact that the region is geographically large and sparsely populated.

Another difference is that the target user group of the STAR-C coach application is all citizens of the region without exception, meaning that individuals who may not conform to a certain norm or culture are included. Commercial applications are typically developed with a target user group in mind, based on personas or other stereotyped user groups to maximize the number of users. Advertisements for health and fitness apps also reinforce the normative images of who the users are anticipated to be or to become, typically good-looking, young, fit, and active individuals running in urban environments. By contrast, the goal of addressing all potential users of the STAR-C coach application is exemplified by the emphasis on how users may define their own “baby-step” goals, instead of pre-defining goals or what would be the norms relating to what counts as steps, running, etc.

### 4.2 On the results informing the design of a person-adapted coaching system for behavior change

It is widely acknowledged that changing behavior is difficult, as a change may only be a temporary inhibition of fundamental habits to which an individual will sooner or later return (36). The fact that changing behaviors is difficult was also a starting point among the participating experts in the domain. Therefore, the design is built on previous experiences of what has been shown to make a change in healthcare, i.e., motivational interviewing, setting goals, person-tailor support, etc. (4, 24). Some norms a person may have internalized on their own, while others they may question and choose to not comply with (37), which may reflect an individual’s readiness for change (23). In both cases, the person makes decisions by taking the norms into account, consciously or unconsciously (38). Consequently, in the moment of deciding whether to act following the plan to increase health behaviors or not, there are a number of factors influencing the decision that the person may not be explicitly taking into consideration, for example, expectations from others, obligations and habits, and priorities relating to time and economy.

Throughout the study, there were shared opinions but also differences in how different participants, including domain experts, viewed the system and its functionality. Therefore, design choices were discussed with arguments in favor and against, and choices were made based on these. While the experts agreed on the functionalities to be included in the system, the various motivators and barriers, some in terms of social norms that could serve as both motivators and barriers, were discussed and there were different views on what to elicit and what to not in the interaction design. Some differences in perspectives were grounded in responsibility and accountability: to what extent can results be promised, when many factors affect a person’s health? One example was how to manage desires to increase physical activity for the purpose of decreasing body weight, which was expressed by users. The domain experts wanted the system to primarily promote health in the short-term perspective through “baby-step goals” and build on small successfully accomplished activities, rather than putting focus on the harder long-term goals. Moreover, weight loss was seen as a particularly hard aim to achieve through a coaching app, so it was decided to not include this as an explicitly termed goal in the application, for which assessment and tailored advice would be generated. Instead, and as a compromise, the user can add this as another self-defined motivation, which they can assess and follow up using the application. Similarly, regarding social constraints that form barriers that hinder users from conducting planned activities, such as others’ expectations, obligations that must be prioritized, or limited economic resources, they can include these in their definitions of activities and in assessing causes when they find that they are not following their plans.

Some examples of norms in the form of arguments were presented to the domain experts and the users through the mock-up and discussed. The participating users elaborated on the social norms, sharing experiences of when social norms relating to others’ expectations prevented them from doing the activities they wanted to do. Viewing social norms as design material was an interesting approach, which we will explore further in future studies. We will also explore how such arguments could be embedded in the dialogues the system has with the user.

Some norms relating to technology and its use were elicited. Participants expected the system and a digital coach to deliver hard facts and be proactive, which would be more acceptable in an application provided by healthcare compared to commercial enterprises. This leads to the questions of what would be appropriate, acceptable, and motivating behavior in a digital health coach in different situations, and how different is this view among different individuals? Studies in the near future will explore this by focusing on the tailored behavior that will be embedded in future versions of the STAR-C application (22).

### 4.3 Strengths and limitations of the study

The main limitation of the empirical study presented in this article is the limited number of participants representing future end users involved in the process so far, with five in the first phase and six in the second phase. The pandemic restrictions played a role but we also recruited participants from among all the citizens taking part in VIP, which also means that individuals with low technology literacy and interest or with limited fluency in the Swedish and/or English languages were asked to participate. Yet, this also shows that digital interventions may not be interesting to a number of citizens taking part in VIP, and the need for interventions that communicate with the person in their preferred language. A common reason for participating in the study was that they wanted to contribute to research and to their regional healthcare. Not all of them were in need of changing their behavior to improve their health; two participants were already actively maintaining healthy habits in the domains targeted in this study, which was the reason for them only participating in the first session in the second phase. As the design and development continues, more participants will be involved.

Another aspect relating to the participatory design methodology is the domain experts acting in different roles during the design process—as researchers, domain experts, representatives of the healthcare organization, clinicians, and even potential end users all typically take part in VIP. Not all were researchers and not all were representatives of the healthcare organization or clinicians. During the design and knowledge engineering process, their different roles were elicited in the sessions regarding the notion of argumentation schemes (20), where design arguments were sorted based on what grounds a design proposal was presented. A strength of adopting the participatory design methodology was that it allowed as many perspectives as possible to be considered in order to elicit potentially conflicting viewpoints, which can cause obstacles later in the process.

In development processes where the users are only given the role of reactive data-providing consumers of products, it is less clear where and how the values that feed into design choices are or could be elicited and debated with users in the design and development process. A strength of adopting the participatory design methodology is that values and norms are elicited. Further, reviewing the process and content from the the perspective of a responsible AI system design methodology shows that AI systems that are aimed to be used for promoting better health can also be targeted using participatory design methods without losing the ethical perspective in the process.

## 5 Conclusions and future work

The purpose of the research presented in this article was to explore the participatory design process of an AI-based digital coaching application for supporting health and preventing cardiovascular diseases from a responsible design of AI systems perspective. The results include increased awareness of the value of participatory design in achieving a value-based design of AI systems aimed at promoting health and the inclusion of social norms as design material in the process. Some open questions were identified and will be addressed in future work. One question is how a STAR-C community could function that allows the individual to support others and receive support from others, while the healthcare-providing organization and its representatives maintain their responsibilities and accountability. Thus, it is a question of how to mitigate adversarial behavior among users.

Other questions relate to how to think about values and norms in the use of the application. What would be the purpose of eliciting norms and allowing users to engage in dialogues about them? In what way do these relate to the individual’s own motivators? This will be explored along the perspective of adopting social norms as design material, which can be used to shape the behavior of an interactive AI system.

It was concluded that what directs which values are manifested in the application is the relationship between the anticipated future users and the organization(s) or enterprises developing and implementing the health-promoting application. The Scandinavian participatory design tradition, with its strong commitment to democratic values through participation, discussions of values, and viewing conflict and contradictions as sources in design, provides an arena where such relationships can also be explored, questioned, and developed.

## Data Availability

The datasets presented in this article are not readily available because the collected qualitative data will not be shared. Requests to access the datasets should be directed to helena.lindgren@umu.se.

## Footnotes

^1^ https://www.myfitnesspal.com/

^2^ https://www.apple.com/ios/health/

## References

[1] Dignum V. Data from: Humane AI ethical framework (2019).

[2] Lindgren H Guerrero E Jingar M Lindvall K Ng N Richter Sundberg L The STAR-C intelligent coach: a cross-disciplinary design process of a behavior change intervention in primary care. Stud Health Technol Inform. (2020) 273:203–8. 10.3233/SHTI200640 33087613

[3] Ng N Eriksson M Guerrero E Gustafsson C Kinsman J Lindberg J Sustainable behavior change for health supported by person-tailored, adaptive, risk-aware digital coaching in a social context: study protocol for the star-c research programme. Front Public Health. (2021) 9:138. 10.3389/fpubh.2021.593453 PMC7957003 33732674

[4] Blomstedt Y Norberg M Stenlund H Nyström L Lönnberg G Boman K Impact of a combined community and primary care prevention strategy on all-cause and cardiovascular mortality: a cohort analysis based on 1 million person-years of follow-up in Västerbotten county, Sweden, during 1990–2006. BMJ Open. (2015) 5:1–9. 10.1136/bmjopen-2015-009651 PMC4691769 26685034

[5] Norberg M Wall S Boman K Weinehall L. The västerbotten intervention programme: background, design and implications. Glob Health Action. (2010) 3:1–15. 10.3402/gha.v3i0.4643 PMC2844807 20339479

[6] Floyd C Mehl W-M Resin F-M Schmidt G Wolf G. Out of scandinavia: alternative approaches to software design and system development. Hum Comput Interact. (1989) 4:253–350. 10.1207/s15327051hci0404/1

[7] Gregory J. Scandinavian approaches to participatory design. Int J Eng Educ. (2003) 19:62–74. http://www.ijee.ie/articles/Vol19-1/IJEE1353.pdf

[8] Coiera E. Putting the technical back into socio-technical system research. Int J Med Inform. (2007) 76:S98–103. 10.1016/j.ijmedinf.2006.05.026 16807084

[9] Bardram JE. Activity-based computing: support for mobility and collaboration in ubiquitous computing. Pers Ubiquitous Comput. (2005) 9:312–22. 10.1007/s00779-004-0335-2

[10] Bodker S. A human activity approach to user interfaces. Hum Comput Interact. (1989) 4:171–95. 10.1207/s15327051hci0403/1

[11] Waern A Back J. Activity as the ultimate particular of interaction design. In: *Proceedings of the 2017 CHI Conference on Human Factors in Computing Systems*. New York, NY, USA: Association for Computing Machinery (2017). CHI ’17. p. 3390–402.

[12] Ehn P. Work-Oriented Design of Computer Artifacts. Stockholm: Arbetslivscentrum (1988).

[13] Friedman B Kahn PH Borning A Huldtgren A. Value Sensitive Design and Information Systems. Dordrecht: Springer Netherlands (2013). p. 55–95.

[14] Friedman B Kahn PH. Data from: Value sensitive design: theory and methods (1992).

[15] Umbrello S. Mapping value sensitive design onto AI for social good principles. AI Ethics. (2021) 1:283–96. 10.1007/s43681-021-00038-3 34790942 PMC7848675

[16] Bratteteig T Verne G. Does AI make pd obsolete? exploring challenges from artificial intelligence to participatory design. In: *Proceedings of the 15th Participatory Design Conference: Short Papers, Situated Actions, Workshops and Tutorial - Volume 2*. New York, NY, USA: Association for Computing Machinery (2018). PDC ’18. doi: 10.1145/3210604.3210646.

[17] Janols R Lindgren H. A method for co-designing theory-based behaviour change systems for health promotion. Stud Health Technol Inform. (2017) 235:368–72. 10.3233/978-1-61499-753-5-368 28423816

[18] Lindgren H Kampik T Rosero EG Blusi M Nieves JC. Argumentation-based health information systems: a design methodology. IEEE Intell Syst. (2021) 36:72–80. 10.1109/MIS.2020.3044944

[19] Paramythis A Weibelzahl S Masthoff J. Layered evaluation of interactive adaptive systems: framework and formative methods. User Model User-Adapt Interact. (2010) 20:383–453. 10.1080/10447318.2021.1925436

[20] Walton D Reed C Macagno F. Argumentation Schemes. Cambridge: Cambridge University Press (2008).

[21] Lindgren H Weck S. Conceptual model for behaviour change progress—instrument in design processes for behaviour change systems. *Stud Health Technol Inform*. To appear (2021). 10.3233/SHTI210614 34734886

[22] Kilic K Weck S Kampik T Lindgren H. Argument-based human-AI collaboration for supporting behavior change to improve health. Front Artif Intell. (2023) 6:1069455. 10.3389/frai.2023.1069455 36872933 PMC9979214

[23] Prochaska J Redding C Evers K. The Transtheoretical Model and Stages of Change. San Francisco: Jossey-Bass/Wiley (2015). p. 97.

[24] Hörnsten Å Lindahl KB Persson KI Edvardsson K. Strategies in health-promoting dialogues–primary healthcare nurses’ perspectives–a qualitative study. Scand J Caring Sci. (2014) 28(2):235–44. 10.1111/scs.12045 23594185

[25] Haggard P. Sense of agency in the human brain. Nat Rev Neurosci. (2017) 18:4196–207. 10.1038/nrn.2017.14 28251993

[26] Limerick H Coyle D Moore JW. The experience of agency in human-computer interactions: a review. Front Hum Neurosci. (2014) 8:643. 10.3389/fnhum.2014.00643 25191256 PMC4140386

[27] Oinas-Kukkonen O Harjumaa M. Persuasive systems design: key issues, process model, and systems features. Commun Assoc Inf Syst. (2009) 24:485–500. 10.17705/1CAIS.02428

[28] van der Velden M Mörtberg C. Participatory Design and Design for Values. Dordrecht: Springer Netherlands (2021). p. 1–22.

[29] Adam MTP Dreyer S Gimpel H Olenberger C. Digital human representations for health behavior change: a structured literature review. AIS Trans Hum Comput Interact. (2022) 14:314–55. 10.17705/1thci.00171

[30] Chan G Nwagu C Odenigbo I Alslaity A Orji R. The shape of mobile health: a systematic review of health visualization on mobile devices. Int J Hum Comput Interact. (2024) 41:1–19. 10.1080/10447318.2024.2313282

[31] Jakob R Harperink S Rudolf AM Fleisch E Haug S Mair JL Factors influencing adherence to mhealth apps for prevention or management of noncommunicable diseases: systematic review. J Med Internet Res. (2022) 24:e35371. 10.2196/35371 35612886 PMC9178451

[32] Laranjo L Quiroz JC Tong HL Arevalo Bazalar M Coiera E. A mobile social networking app for weight management and physical activity promotion: results from an experimental mixed methods study. J Med Internet Res. (2020) 22:e19991. 10.2196/19991 33289670 PMC7755540

[33] Berdichevsky D Neuenschwander E. Towards an ethics of persuasive technology. Commun ACM. (1999) 42:51–8. 10.1145/301353.301410

[34] Kampik T Nieves JC Lindgren H. Coercion and deception in persuasive technologies. In: *Proceedings of the 20th International Trust Workshop, CEUR-WS, Stockholm*. (2018). p. 38–49.

[35] Tengland P. Behavior change or empowerment: on the ethics of health-promotion goals. Health Care Anal. (2016) 24:24–46. 10.1007/s10728-013-0265-0 24100936

[36] Bouton ME. Why behavior change is difficult to sustain. Prev Med. (2014) 68:29–36. 10.1016/j.ypmed.2014.06.010 24937649 PMC4287360

[37] Ryan R Deci L. Self-determination theory and the facilitation of intrinsic motivation, social development and well-being. Am Psychol. (2000) 55:68–78. 10.1037/0003-066X.55.1.68 11392867

[38] Kahneman D. Thinking Fast and Slow. London: Penguin Books (2017).

